# Patient Concerns Regarding Artificial Intelligence Applications in Health Care: Systematic Review and Meta-Synthesis Based on Social Ecological Theory

**DOI:** 10.2196/85663

**Published:** 2026-04-28

**Authors:** Jiayu Hou, Zhiqiao Zhang, Xuan Cheng, Weihong Wang

**Affiliations:** 1 School of Nursing, Hunan Normal University, No. 371, Tongzipo Road, Yuelu District, Changsha City, Hunan Province, China, 86 13548968918; 2 Kiang Wu Nursing College of Macau, Macao, China; 3 Medical Humanities Research Center, Hunan Normal University, Changsha, China

**Keywords:** artificial intelligence, medical ethics, patient concerns, data privacy, physician-patient relationship, health care equity, meta-integration, social ecological theory

## Abstract

**Background:**

The use of artificial intelligence (AI) in health care is growing quickly, but there is not enough research that looks at patient concerns from a multilevel perspective. Existing reviews predominantly summarize patient attitudes descriptively, lacking theoretical frameworks to explain the underlying mechanisms of these concerns.

**Objective:**

This systematic review and meta-synthesis aimed to identify and analyze patient concerns regarding health care AI applications, using social ecological theory to reveal the multilevel interactive mechanisms of concern at the individual, interpersonal, organizational, and societal levels.

**Methods:**

Following the PRISMA-S (Preferred Reporting Items for Systematic Reviews and Meta-Analyses literature search extension) guidelines, databases including PubMed, Embase, Web of Science, CINAHL, and Scopus were searched on March 1, 2026. Qualitative studies exploring patient perceptions of clinical AI applications were included, excluding those involving only healthy populations, technical performance, or nonclinical settings. Two researchers independently screened the literature and assessed methodological quality using the JBI-QARI (Joanna Briggs Institute Qualitative Assessment and Review Instrument) checklist. Confidence in synthesized findings was assessed using the GRADE-CERQual (Confidence in the Evidence from Reviews of Qualitative Research) approach.

**Results:**

A total of 25 qualitative studies involving 528 participants from diverse patient groups across multiple countries were included. Six themes emerged: (1) microlevel worries about privacy and data security, including data breaches and loss of control over personal health information; (2) worries about the limits and reliability of technology, especially AI diagnostic accuracy and “black box” decision-making; (3) mesolevel effects on physician-patient relationships, including reduced face-to-face interaction and empathy; (4) trust and accountability issues, including unclear responsibility attribution and institutional oversight problems; (5) macrolevel ethical and equity issues, including algorithmic bias and health care access inequalities; and (6) worries about technology diffusion and possible replacement of health care workers.

**Conclusions:**

This review represents the first meta-synthesis applying social ecological theory to construct patient concerns regarding medical AI. Unlike previous descriptive reviews, it reveals the interconnected “ecological imbalance” mechanisms at micro-, meso-, and macrolevels when AI is embedded in health care systems. The findings suggest that many patient concerns are based on facts rather than just misunderstandings, indicating that systemic rather than isolated interventions are needed. Practical implications include explainable algorithm design at the microlevel, improved physician-patient communication, and institutional accountability at the mesolevel, and coordinated global ethical norms and equity-promoting policies at the macrolevel. Limitations include the inclusion of studies primarily from developed regions, significant heterogeneity in AI application scenarios, and constraints inherent to secondary research. Nevertheless, addressing these multilevel concerns remains crucial for balancing technological advancement with patient-centered care and enabling sustainable AI integration.

## Introduction

The application of artificial intelligence (AI) technology in health care is rapidly advancing, particularly across multiple domains, including disease diagnosis, treatment decision support, personalized health management, image analysis, and drug discovery [1]. Through techniques such as deep learning and natural language processing, AI can extract critical insights from vast datasets, empowering clinicians to make more precise decisions. With breakthroughs in generative AI and large language models, AI capabilities have expanded into complex clinical decision support and physician-patient interactions, enhancing communication and improving patient outcomes through more tailored and responsive care [2]. As of 2024, the US Food and Drug Administration has approved over 950 AI or machine learning medical devices, with radiology accounting for 76% of these approvals [3]. In 2024, AI adoption among US physicians reached 66%, nearly doubling from 38% in 2023 [4]. AI implementation has significantly enhanced health care efficiency, optimized resource allocation, and delivered more personalized and precise treatment plans for patients [5]. For instance, AI can identify minute lesions during early screening and match genes with therapies in precision medicine, substantially improving cure rates and patient quality of life [6]. However, as AI technology becomes more widespread and deeply integrated into health care, patient concerns regarding ethics, privacy, and security have increasingly come to the fore [7]. These concerns not only impact patient acceptance of AI technology but also directly affect the smooth promotion and implementation of AI in medical practice [8].

When encountering AI-based medical applications, one of the patients’ primary concerns is privacy protection and data security [9]. With the digitization of medical data and widespread AI adoption, patient health information is being collected, stored, and analyzed at an unprecedented scale. Many patients fear potential misuse or leakage of these sensitive data, particularly when data protection measures remain inadequate [10]. A 2023 national survey revealed that nearly half of US adults express low trust in the health care system’s responsible use of AI [11]. Additionally, the transparency and explainability of AI decisions have become major patient concerns. Due to the “black box” nature of AI algorithms, patients often cannot understand how AI arrives at diagnostic and treatment recommendations. This opacity fuels skepticism about the reliability of AI systems [12] and hinders clinicians’ ability to explain treatment decisions to patients [13]. Another core issue is the potential loss of patient decision autonomy, particularly in urgent situations such as emergency rooms. AI’s “automated” decisions may deprive patients of choice and diminish their active role in the treatment process [14]. Furthermore, as AI gradually assumes certain medical tasks, patient concerns about the erosion of humanistic care are growing. Many patients believe that while AI can provide efficient diagnostic support, it cannot replace a physician’s empathy and care. The resulting distancing in the physician-patient relationship may impact treatment outcomes and the overall patient experience [15]. A mixed methods survey of 600 US adults revealed that 30% of respondents expressed concern about AI’s lack of a physician’s “human touch,” while 84.2% preferred AI for tasks unrelated to the physician-patient relationship, such as appointment scheduling [16].

Therefore, the application of AI in health care involves not only technological advancement but also profound shifts in physician-patient relationships, ethical principles, and cultural values. During the development of new technologies, it is important to fully consider and address patients’ ethical concerns, need for privacy protection, and expectations for more humanized medical care. Existing qualitative syntheses primarily adopt a broad public perspective, empirically summarizing the advantages, risks, and recommendations of AI in health care [17], yet theoretical frameworks remain relatively underdeveloped. Given this context, this study aimed to synthesize existing literature systematically on patients’ ethical concerns regarding AI through the Social Ecological Model framework. Specific research objectives are as follows:

1. To identify fundamental ethical issues (eg, privacy, trust, and humanistic care) that patients encounter with AI health care applications;
2. To analyze how these concerns influence patient acceptance of AI technology;
3. To reveal tension mechanisms at 3 levels—individual cognition, physician-patient interaction, and macrolevel institutional systems—to address potential “ecological imbalance risks.”

## Methods

### Study Registration and Reporting Framework

This study strictly followed the PRISMA (Preferred Reporting Items for Systematic Reviews and Meta-Analyses) guidelines (Checklist 1) [18] and the ENTREQ (Enhancing Transparency in Reporting the Synthesis of Qualitative Research; Checklist 2) [19]. This review aimed to explore patients’ values, attitudes, and experiences regarding the application of AI in health care. The Cochrane Handbook recognizes qualitative evidence synthesis as the appropriate methodology for such questions [20]. As all 25 included studies used qualitative designs yielding narrative findings rather than quantitative effect estimates amenable to statistical pooling [21], statistical meta-analysis was not applicable to this review. We therefore used the Joanna Briggs Institute meta-aggregation approach [22]. The review was prospectively registered in PROSPERO (CRD420251156502). The registered protocol was subsequently updated to clarify the qualitative study design and the use of JBI-QARI (Joanna Briggs Institute Qualitative Assessment and Review Instrument) for quality assessment. In addition, the following methodological enhancements were made during the conduct of this review: (1) CINAHL was added as a fifth database to improve coverage of nursing and allied health literature, (2) the GRADE-CERQual (Confidence in the Evidence from Reviews of Qualitative Research) approach was adopted to assess confidence in the synthesized findings, and (3) social ecological theory (SET) was used as a theoretical lens to interpret and discuss the findings.

### Search Strategy

This study’s search strategy followed the PRISMA-S (Preferred Reporting Items for Systematic Reviews and Meta-Analyses literature search extension) guideline (Checklist 3) [23]. The initial search was conducted on September 28, 2025. Following iterative refinement of the search strategy, updated searches were performed on January 4, 2026, and March 1, 2026, to capture the most recent literature. Five databases were searched from inception to the date of each search: PubMed (via National Library of Medicine), Embase (via Embase.com), Web of Science (via Clarivate, encompassing the Core Collection, KCI-Korean Journal Database, MEDLINE, ProQuest Dissertations & Theses Citation Index, SciELO Citation Index, and the Grants Index), CINAHL (via EBSCOhost), and Scopus (via Scopus.com); all other databases were searched independently through their respective platforms. The detailed search strategies for all databases are provided in Multimedia Appendix 1. A 3-step search strategy was used. First, an initial search was conducted in PubMed, and the titles, abstracts, and index terms of relevant records were analyzed to identify key search terms. Second, a comprehensive search using all identified keywords and index terms was undertaken across all databases. Third, the reference lists of included studies were hand-searched to identify any additional relevant studies. The search strategy was developed de novo for this review, and its effectiveness was validated by confirming the retrieval of known relevant studies. No formal peer review of the search strategy was conducted using standardized appraisal tools. This review focused on published qualitative research; clinical trial registries were not searched. Beyond the systematic database searches and reference list screening, no supplementary search methods were used, such as contacting authors, browsing conference proceedings, or setting up citation alerts. Only studies published in English or Chinese were included. The PubMed search strategy is presented below:

- #1 (“Artificial Intelligence”[MeSH]) OR (“Machine Learning”[MeSH]) OR (“Decision Support Systems, Clinical”[MeSH]) OR (“artificial intelligence”[tiab]) OR (“machine learning”[tiab]) OR (“deep learning”[tiab]) OR (“AI-based”[tiab]) OR (“AI-assisted”[tiab]) OR (“AI-driven”[tiab]) OR (“ChatGPT”[tiab]) OR (“large language model”[tiab]) OR (“clinical decision support”[tiab]) OR (“CDSS”[tiab]) OR (“generative AI”[tiab]) OR (“algorithm*"[tiab])
- #2 (“Patients”[MeSH]) OR (patient*[tiab]) OR (stakeholder*[tiab])
- #3 (healthcare[tiab]) OR (“health care”[tiab]) OR (medical[tiab]) OR (clinical[tiab])
- #4 (“Attitude to Health”[MeSH]) OR (concern[tiab]) OR (perception[tiab]) OR (perspective[tiab]) OR (attitude[tiab]) OR (trust[tiab]) OR (acceptance[tiab]) OR (barrier[tiab]) OR (ethical[tiab]) OR (privacy[tiab]) OR (view[tiab]) OR (opinion[tiab]) OR (experience[tiab]) OR (feeling[tiab]) OR (worry[tiab])
- #5 (“Qualitative Research”[MeSH]) OR (qualitative[tiab]) OR (“focus group”[tiab]) OR (interview[tiab]) OR (“thematic analysis”[tiab]) OR (“content analysis”[tiab]) OR (“grounded theory”[tiab]) OR (phenomenolog*[tiab]) OR (ethnograph*[tiab]) OR (“lived experience”[tiab]) OR (“narrative analysis”[tiab]) OR (“in-depth interview”[tiab]) OR (“qualitative study”[tiab]) OR (“qualitative research”[tiab])
- #6 #1 AND #2 AND #3 AND #4 AND #5

### Inclusion and Exclusion Criteria

This review focused on patient concerns arising from the use of AI in clinical practice. The inclusion and exclusion criteria are presented in Table 1. A total of 19,090 records were retrieved and imported into EndNote (version 21; Clarivate Analytics). Through both automated and manual deduplication in EndNote 21, a total of 7132 (37.4%) duplicate records were identified, leaving 11,958 (62.6%) records for assessment based on the relevance of titles and abstracts. At the title and abstract screening stage, 11,874 (99.3%) records were excluded for the following reasons: not addressing AI applications in clinical health care settings (n=5818, 49%), not involving patients as participants (n=3871, 32.6%), ineligible study design (n=2030, 17.1%), and not published in English or Chinese (n=155, 1.3%). A total of 84 (0.7%) records met the selection criteria, and their full texts were retrieved for further evaluation. Following full-text assessment, 25 studies met the criteria for quality appraisal. Two authors independently conducted the screening process, and any disagreements regarding inclusion were resolved through consultation with a third author.

**Table 1.** Inclusion and exclusion criteria for qualitative studies on patient concerns regarding artificial intelligence (AI) applications in health care based on the PICOS framework.

Components	Inclusion criteria	Exclusion criteria
Participants	Patients who receive medical services supported by AI technology and stakeholders.	Stakeholders without patients and healthy people.
Phenomenon of interest	The focus of the research is on the application of AI technology in health care, such as AI-assisted diagnosis, AI-assisted surgeries, AI health monitoring, and virtual nursing assistants. The research focuses on patients’ perception of AI applications, their psychological reactions, concerns, and emotions, such as concerns about privacy, fears of physician substitution, and trust in AI.	It does not involve the application of AI technology in medical fields, or does not focus on the research of patients’ psychological and emotional responses. Only focusing on the technical aspects such as the performance and algorithm optimization of AI technology, without considering the patients’ perception and emotions.
Context	The research background focuses on AI application scenarios in the medical environment, including hospitals, clinics, telemedicine platforms, and public health monitoring.	Research that is not conducted in actual medical settings or that is limited to technological development research in laboratory environments. The research environment has no relation to the actual medical experience of patients and thus cannot provide in-depth information about patients’ perceptions and reactions.
Study design	The data collection method focuses on the subjective experiences of patients, paying attention to their emotional, cognitive, and worrying psychological reactions during their medical intervention with AI technology.	Not a qualitative study. The overly simplistic design, such as relying solely on short questionnaires or tool tests, fails to thoroughly explore the psychological and emotional responses of patients.

### Quality Assessment

The JBI-QARI was used to evaluate the methodological rigor of each published study [24]. Questions answered “yes” received 1 point, and studies scoring 5 points or lower were deemed low quality and excluded from the synthesis. Two reviewers independently conducted rigorous assessments of the selected research reviews. Disagreements were resolved through discussion or consultation with a third reviewer within the team. Table 2 presents the quality assessment results for the studies included in this review.

**Table 2.** Methodological quality assessment of 25 included qualitative studies using the Joanna Briggs Institute Qualitative Assessment and Review Instrument critical appraisal checklist.

Studies	Q1^a^	Q2^b^	Q3^c^	Q4^d^	Q5^e^	Q6^f^	Q7^g^	Q8^h^	Q9^i^	Q10^j^	Total
Hurley et al [25]	Y^k^	Y	Y	Y	Y	Y	U	Y	Y	Y	9
Annamalai [26]	U	Y	Y	Y	Y	U	N^l^	Y	Y	Y	7
Čartolovni et al [27]	Y	Y	Y	Y	Y	U^m^	N	Y	Y	Y	8
Sujan et al [28]	Y	Y	Y	Y	Y	U	N	Y	Y	Y	8
Kostick-Quenet et al [29]	Y	Y	Y	Y	Y	Y	U	Y	Y	Y	9
Hesjedal et al [30]	Y	Y	Y	Y	Y	N	Y	Y	Y	Y	9
McCradden et al [31]	Y	Y	Y	Y	Y	U	U	Y	Y	Y	8
Al-Anezi [32]	Y	Y	Y	Y	Y	U	U	Y	Y	Y	8
Freeman et al [33]	Y	Y	Y	Y	Y	U	U	Y	Y	Y	8
Viberg Johansson et al [34]	Y	Y	Y	Y	Y	N	U	Y	Y	Y	8
Jeyakumar et al [35]	Y	Y	Y	Y	Y	N	U	Y	Y	Y	8
Haan et al [36]	U	Y	Y	Y	Y	U	U	Y	Y	Y	7
Berger et al [37]	Y	Y	Y	Y	Y	U	U	Y	Y	Y	8
Khairat et al [38]	Y	Y	Y	Y	Y	U	U	Y	Y	Y	8
Litchfield et al [39]	U	Y	Y	Y	Y	U	U	Y	Y	Y	7
Omori et al [40]	Y	Y	Y	Y	Y	U	U	Y	U	Y	7
Funer et al [41]	U	Y	Y	Y	Y	N	U	Y	Y	Y	7
Giebel et al [42]	U	Y	Y	Y	Y	Y	Y	Y	Y	Y	9
Ly et al [43]	Y	Y	Y	Y	Y	Y	Y	Y	Y	Y	10
Richardson et al [44]	N	Y	Y	Y	Y	U	U	Y	Y	Y	7
Foresman et al [45]	Y	Y	Y	Y	Y	U	U	Y	Y	Y	8
Schneider et al [46]	N	Y	Y	Y	Y	N	N	Y	Y	Y	7
Zhang et al [47]	U	Y	Y	Y	Y	N	U	Y	Y	Y	7
Gundlack et al [48]	U	Y	Y	Y	Y	N	U	Y	Y	Y	7
Steerling et al [49]	U	Y	Y	Y	Y	U	Y	Y	Y	Y	8

a Q1. Is there congruity between the stated philosophical perspective and the research methodology?

b Q2. Is there congruity between the research methodology and the research question or objectives?

c Q3. Is there congruity between the research methodology and the methods used to collect data?

d Q4. Is there congruity between the research methodology and the representation and analysis of data?

e Q5. Is there congruity between the research methodology and the interpretation of results?

f Q6. Is there a statement locating the researcher culturally or theoretically?

g Q7. Is the influence of the researcher on the research, and vice-versa, addressed?

h Q8. Are participants, and their voices, adequately represented?

i Q9. Is the research ethical according to current criteria or, for recent studies, and is there evidence of ethical approval by an appropriate body?

j Q10. Do the conclusions drawn in the research report flow from the analysis, or interpretation, of the data? the conclusions drawn in the research report flow from the analysis, or interpretation, of the data?

k Y: yes.

l N: no.

m U: unclear.

### Data Extraction and Synthesis

Data were extracted from the studies included in this review using the JBI-QARI standardized data extraction tool. The first author extracted data from these 25 studies, including the first author and publication year, study population and sample size, research topic, study type, and primary outcomes, as detailed in Table 3. This study used a meta-synthesis approach [22]. Two researchers repeatedly read and interpreted the original studies, analyzed and interpreted the implications of the findings, grouped similar results into new categories, and then synthesized these categories into integrated outcomes to form new perspectives or interpretations. When disagreements arose between the 2 coders, a third coder was consulted.

The confidence in each synthesized finding was assessed using the GRADE-CERQual approach [50]. CERQual assesses confidence based on four components: (1) methodological limitations of the included studies, informed by the JBI-QARI critical appraisal results; (2) coherence of the finding across contributing studies; (3) adequacy of data supporting the finding, considering both the number of studies and the richness of data; and (4) relevance, defined as the extent to which the contexts of contributing studies are applicable to the review question. Each finding was assigned an overall confidence level of high, moderate, low, or very low. Two reviewers independently assessed each component and resolved disagreements through discussion. The CERQual assessment results are presented in Table 4. Heterogeneity across included studies was explored narratively by examining differences in AI application types, patient populations, and geographic contexts, as reported in Table 3 and discussed in the *Limitations* section.

**Table 3.** Characteristics of 25 included qualitative studies on patient concerns regarding artificial intelligence (AI) in health care (2019‐2025).

Studies	Year	Country	Setting	Data collection	Research method	Participants	Interested topics	Main results
Hurley et al [25]	2024	United States	Not specified	Semistructured interviews	Phenomenological research	Stakeholders (including 20 patients)	Ethical considerations in integrating multimodal computer perception and neurotechnology in clinical care	Three main themes: Perceived invasiveness of passive and continuous data collection Data protection and security concerns Ethical issues related to patients’ awareness of data collection
Annamalai [26]	2020	India	Telepsychiatry	Semistructured interviews	Grounded theory approach	Stakeholders (including 14 patients)	Exploring challenges of AI-enabled telepsychiatry for clinical practice among urban Indian stakeholders	Four themes: Ethical, legal, accountability, and regulatory issues Challenges related to data Health system infrastructure AI related
Čartolovni et al [27]	2023	Croatia	Hospital	Semistructured interviews	Phenomenological research	Stakeholders (including 15 patients)	Exploring multistakeholder (patients, physicians, and health care managers) insights into AI’s impact on the patient-physician relationship	Four themes: The current state of health care and the patient-physician relationship Expectations of AI a synergetic effect between physicians AI the future of health care The patient-physician relationship
Sujan et al [28]	2022	United Kingdom	Hospital	Semistructured interviews	Phenomenological research	Stakeholders (including 4 patients)	Exploring perceptions of safety and safety assurance of health care AI (using AI-based ICU^a^ infusion pumps as a case) among patients, hospital staff, technology developers, and regulators in the United Kingdom	Four themes: The potential impact of health care AI Requirements for human-AI interaction Safety assurance practices and regulatory frameworks for AI and the gaps that exist How incidents involving AI should be managed
Kostick-Quenet et al [29]	2024	United States	Cardiology	Semistructured interviews	Phenomenological research	Stakeholders (including 18 patients)	Exploring trust criteria for an AI/ML^b^-based survival prediction algorithm (for LVAD^c^ therapy in advanced heart failure) among patients, nurse coordinators, and physicians, focusing on normative and epistemic considerations	Three themes: Epistemic trust considerations Relational trust considerations Personal belief-based trust considerations
Hesjedal et al [30]	2024	Norway	Prostate cancer diagnostics	Participant observation, semistructured interviews, and focus groups	Phenomenological research	Stakeholders (including 38 patients)	Investigating how scientists, MDs, and patients with PCa^d^ relate ethical challenges of AI decision-making tools in PCa diagnostics to their understanding of “good health care,” focusing on registers of valuing	The main finding of the study is that medical physicians, scientists, and patients perceive ethical challenges with AI in prostate cancer diagnostics differently, shaped by their roles and experiences, which influence their understanding of good health care.
McCradden et al [31]	2020	Canada	Hospital	Semistructured interviews	Phenomenological research	30 patients and caregivers	Investigate perspectives on ethical issues surrounding AI in health care research among Canadian patients with meningioma, their caregivers, and health care providers	Eight themes: Consent Privacy Confidentiality Responsibility Accountability Unintended consequences/harms Trust Public engagement
Al-Anezi [32]	2024	Saudi Arabia	Chronic disease management	Semistructured interviews	Phenomenological research	29 patients	Analyze the feasibility of ChatGPT (free version 3.5) as a virtual health coach for chronic disease management, focusing on its ability to promote health literacy and support patients’ self-management	The main finding of this study is that ChatGPT shows potential as a virtual health coach by providing accessible lifestyle advice and motivation, but it faces limitations in accuracy, personalization, and trust that must be addressed before clinical use.
Freeman et al [33]	2024	Australia	Emergency department	Semistructured interviews	Grounded theory approach	28 patients	Health consumers’ ethical concerns toward the use of AI in Australian emergency departments.	Six themes: Health consumer autonomy and consent Physician decision-making and autonomy Automation bias and reliance Data use and privacy Bias and discrimination Regulation
Viberg Johansson et al [34]	2024	Sweden	Breast cancer screening	Semistructured interviews	Phenomenological research	16 patients	Swedish women’s perceptions and attitudes toward the use of AI in mammography (as part of the national breast cancer screening program)	Three themes: Perceived differences between AI and human assessment Attitudes when implementing AI in mammography Requirements when using AI in mammography
Jeyakumar et al [35]	2023	Canada	Acute or long-term medical centers	Semistructured interviews	Phenomenological research	12 patients	How patients view AI in health care and emphasize the need for trust, engagement, and strong data governance to ensure ethical and effective integration	Three themes: Cultivating patients’ trust fostering Patient engagement establishing data governance Validation of AI technologies
Haan et al [36]	2019	Netherlands	Department of Radiology at a tertiary care academic institution	Semistructured interviews	Grounded theory approach	20 patients	Patient perspective on the use of AI in radiology, including awareness, uncertainties, and expectations	Six key domains: Proof of technology (need for evidence on efficacy and reliability of AI) Procedural knowledge (understanding how AI is implemented in practice) Competence (capability of AI to produce reliable results) Efficiency (faster scanning and reduced waiting times) Personal interaction (importance of human communication for results) Accountability (responsibility for errors made by AI)
Berger et al [37]	2025	Norway	Prostate cancer diagnostics (AI supported)	Semistructured interviews	Phenomenological research	18 patients	Patient perspectives on trust in AI-powered tools in prostate cancer diagnostics	Three main dimensions of trust were identified: Foundational trust Interpersonal trust as mediator Need for human oversight additional finding: participants were more forgiving of human errors than those made by AI, highlighting the relational and moral dimensions of trust in health care.
Khairat et al [38]	2025	United States	Cancer survivor organizations (recruitment); virtual/zoom (interviews)	Semistructured interviews	Phenomenological research	21 patients	Cancer survivors’ experiences, facilitators, and barriers regarding the use of AI-based conversational tools (chatbots)	Three overarching themes: Preference for humans: participants strongly preferred interacting with health care professionals over chatbots. Lack of empathy: chatbots were perceived as lacking the necessary empathy and emotional support. Information and privacy concerns: concerns about information overload (generic and nonspecific responses) and data privacy.
Litchfield et al [39]	2025	United Kingdom	Primary care	Semistructured interviews	Phenomenological research	7 patients	Acceptability of “AmarDoctor,” an AI-enabled translation and symptom-checking tool, among underserved Bangladeshi populations	Three overarching themes: Enhanced accessibility and inclusivity Anonymity for sensitive issues Trust and safety concerns
Omori et al [40]	2025	Australia	Screening program	Semistructured interviews and focus groups	Grounded theory approach	26 patients	Development of a typology of women’s attitudes toward the use of AI in breast cancer screening	Four attitude types identified: Enthusiasts Practicalists Traditionalists Guardians
Funer et al [41]	2024	Germany	Surgery, nephrology, intensive home care	Semistructured interviews and focus groups	Phenomenological research	Stakeholders (including 18 patients)	Impacts of CDSSs^e^ on the relationship, communication, and shared decision-making	Three overarching themes: Impact on professional roles Impact on relationship Transparency needs
Giebel et al [42]	2025	Germany	Hospital	Semistructured interviews	Phenomenological research	Stakeholders (including 4 patients)	Opportunities to optimize AI-based CDSS and their integration into health care	Three overarching themes: System optimization User competence and support Environmental framework
Ly et al [43]	2025	Australia	Ophthalmology	Semistructured interviews	Phenomenological research	Stakeholders (including 8 patients)	Stakeholder experiences, attitudes, enablers, barriers, and possible futures of digital diagnosis using AI for age-related macular degeneration in Australia	Three overarching themes: Technological preferences Divergent stakeholder priorities Systemic and ethical considerations
Richardson et al [44]	2021	United States	Primary care	Focus groups	Phenomenological research	87 patients	Patient apprehensions and perspectives regarding the use of AI in health care	Major themes identified: Safety and human oversight Preservation of choice Cost, equity, and data concerns: cost, bias, security
Foresman et al [45]	2025	United States	Primary care, radiology, telehealth	Focus groups	Phenomenological research	17 patients	Patient perspectives on AI use in health care, specifically in diagnostic processes and communication	Five cross-cutting themes emerged: Validation Usability Transparency Opportunities, privacy, additional finding: comfort levels varied by interaction type; participants were most comfortable with “Digital Scribe” (low interaction) and least comfortable with “virtual human” (high interaction).
Schneider et al [46]	2025	Germany	Nephrology, surgery, home-ventilated care	Focus groups	Phenomenological research	18 patients	Patient perspectives on AI-based AI-CDSS, specifically focusing on trust, responsibility, and self-determination	Three overarching observations: Indecision and uncertainty Shift in trust and responsibility AI as support, not replacement
Zhang et al [47]	2021	United States	Radiology	Semistructured interviews	Phenomenological research	13 patients	Patients’ perceptions and acceptance of using AI-based technology to interpret and comprehend radiology reports or imaging data	Four main findings: Positive attitude and utility Concerns Trust and design requirements Designers must ensure systems deliver concerning health results in an empathetic manner to optimize user experience.
Gundlack et al [48]	2025	Germany	Primary care and psychiatry	Semistructured focus groups	Phenomenological research	35 patients	Patients’ perceptions of AI in medical care regarding caregiving relationships and ethics	Four main themes: AI perceived as beneficial for efficiency, data processing, and patient safety. Key concerns include impersonality, data security, and overreliance on AI by medical staff. Human interaction and emotional understanding deemed irreplaceable by AI. Physicians considered primarily responsible for AI-related decisions; data transparency and privacy protection are essential for implementation.
Steerling et al [49]	2025	Sweden	Primary care	Semistructured interviews	Phenomenological research	14 health care professionals and 12 patients	Influences on trust in the use of AI-based triage in primary care	Three main influences on trust: Provision of accurate patient information (patients’ capability and willingness). Alignment with clinical expertise (standardized reasoning and experience-based knowledge). Supervision of patients’ health and safety (professionalism and guidance in information use). Both groups emphasized constructive dialogue and clear instructions for the use and storage of information.

a ICU: intensive care unit.

b ML: machine language.

c LVAD: left ventricular assist device.

d PCa: prostate cancer.

e CDSS: clinical decision support system.

**Table 4.** GRADE-CERQual summary of qualitative findings on patient concerns regarding artificial intelligence in health care.

Review finding	Contributing studies	Methodological limitations	Coherence	Adequacy	Relevance	Overall confidence
Theme 1 (3.1): Privacy and data security	13 studies	Minor concerns	No concerns	No concerns	No concerns	High
Theme 2 (3.2): Technical limitations and reliability	11 studies	Minor concerns	No concerns	No concerns	No concerns	High
Theme 3 (3.3): Impact on physician-patient relationship	11 studies	Minor concerns	No concerns	No concerns	No concerns	High
Theme 4 (3.4): Trust and accountability	13 studies	Minor concerns	No concerns	No concerns	No concerns	High
Theme 5 (3.5): Ethics and equity	8 studies	Minor concerns	No concerns	Minor concerns	Moderate concerns	Moderate
Theme 6 (3.6): Future outlook	10 studies	Minor concerns	No concerns	No concerns	No concerns	High

## Results

### Overview

**Figure 1.**
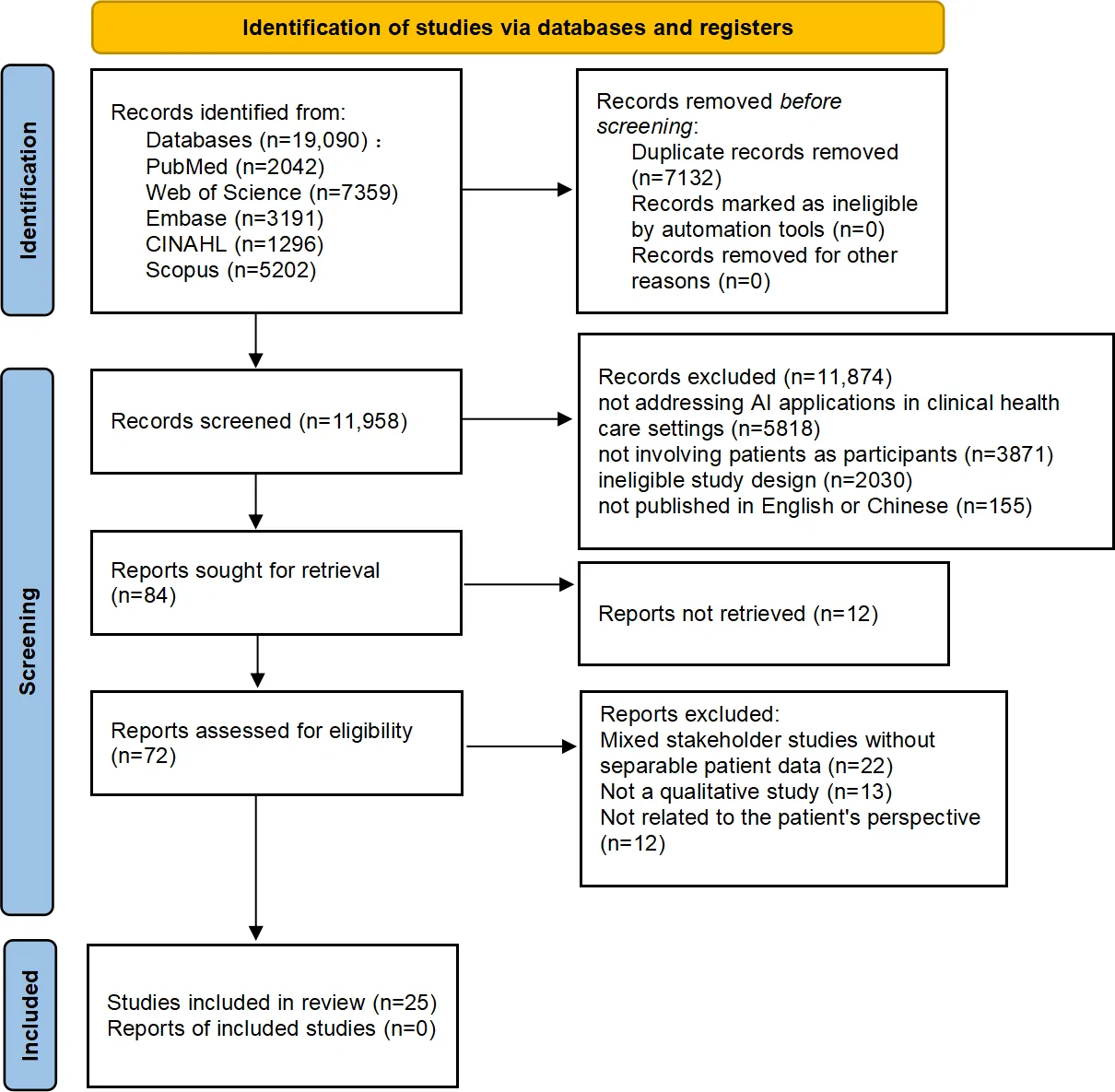
PRISMA (Preferred Reporting Items for Systematic Reviews and Meta-Analyses) flow diagram of study selection process (5 databases, inception to March 2026).

**Figure 2.**
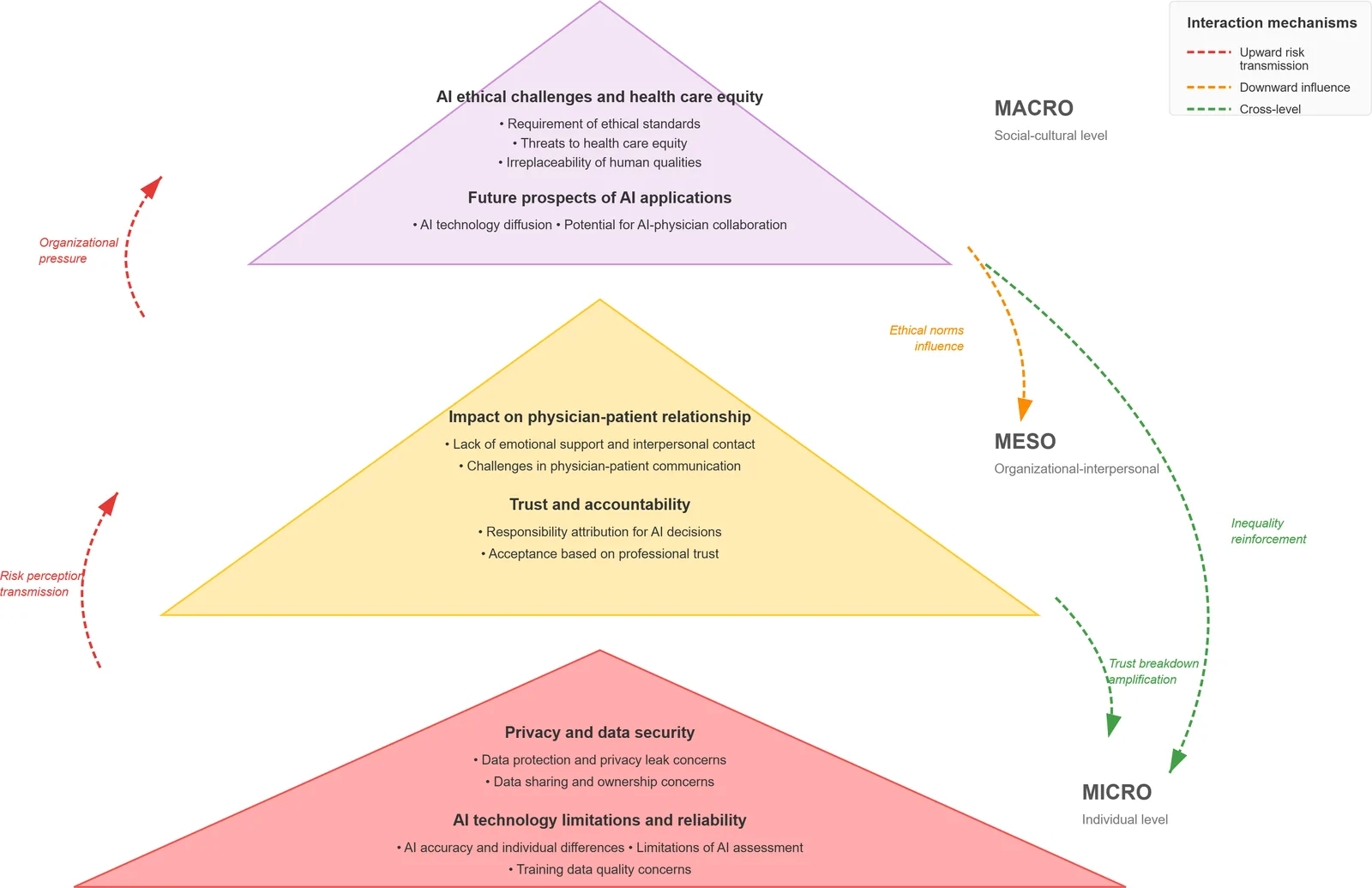
Social ecological theory of patient concerns regarding artificial intelligence (AI) in health care: thematic structure and multilevel interactions (25 studies, n=528).

This paper provides a systematic review and qualitative synthesis of ethical issues surrounding the application of AI in health care. A total of 25 qualitative studies involving 528 patients were included. Details of the search and screening process are presented in the PRISMA flow diagram (Figure 1). The findings were categorized into 14 themes and further consolidated into 6 key themes (Figure 2), elaborated as follows.

### Privacy and Data Security

#### Concerns Over Data Protection and Privacy Breaches

Patients express concerns about the potential misuse of collected medical data, particularly in the absence of effective safeguards. Many believe that once data are uploaded to the internet, they cannot be completely erased and may even be exploited for commercial purposes. Some patients state they consent to data use only for medical purposes, provided they are explicitly informed of its scope:

*They're going to use that [data] for whatever, and [it’s like] the Internet, when you [post] something [and] it’s stuck forever [there].*

[P_05] [25]

*I am not confident enough in sharing my personal health data with ChatGPT, as it is based on AI, and there is a high possibility that the data could be misused.*

[Unspecified] [32]

#### Concerns Regarding Data Sharing and Ownership

Patients expressed confusion about data ownership, particularly when they were unclear about how their data were shared or used. They desire explicit prior notification before data use and fear unauthorized third-party access. Some patients demanded assurances that data use would not exceed the scope of their informed consent:

*I would like to know beforehand so that they don't just send it off without informing me. It doesn't feel entirely right.*

[P_08] [34]

*I'm worried that the data might be used by others without my permission, especially when I don't know the full extent of how it will be used.*

[P_14] [25]

### Limitations and Reliability of AI Technology

#### AI Accuracy and Individual Variability

Patients generally believe that while AI algorithms can analyze vast amounts of data, they cannot fully adapt to each individual’s unique health condition. A patient’s health status is influenced by factors such as genetics, lifestyle, and environment—variables that algorithms often cannot fully predict. Particularly in the early stages of disease or complex scenarios, AI may fail to capture these individual differences, resulting in assessments that do not precisely align with each patient’s specific needs. Therefore, patients view AI primarily as a reference tool, asserting that final diagnostic and treatment decisions should remain with experienced physicians:

*The accuracy would be kind of iffy, because everybody has their own risk factor...*

[P_09] [29]

*I'm fully aware that everybody’s situation is unique to their own individual set of circumstances...*

[P_03] [29]

#### Limitations of AI Assessment

Although AI can process vast amounts of information and provide data-driven analysis, patients believe it cannot comprehensively account for all factors potentially affecting health. For instance, AI struggles to anticipate the impact of nonroutine variables such as unexpected events, environmental changes, or sudden health issues. Patients noted that AI assessments are typically built on historical data, failing to dynamically reflect current health status or psychological state. This limitation frequently constrains AI assessments to broad contexts. Consequently, patients view AI assessments as supplementary tools rather than complete replacements for traditional physician judgment:

*There are so many variables that you don't understand or can't predict...*

[P_11] [29]

*A lot of these assessments aren't taking things into consideration like diabetes, exercise levels...*

[P_04] [29]

#### Concerns Over Training Data Quality

Patients’ doubts about AI reliability extend beyond the algorithms themselves to the quality of their training data. They worry that medical AI systems’ performance heavily depends on the data they learn from, yet real-world electronic health records often suffer from incomplete documentation, outdated information, or human input errors. If AI is trained on such flawed data, even the most sophisticated algorithms struggle to produce accurate, reliable outputs. Patients are further unsettled by the invisible risk of “garbage in, garbage out”—they cannot know what data the AI has learned from, nor whether it accurately reflects patients’ actual health conditions:

*So I've had a lot of different things in my medical chart that are inaccurate, very inaccurate, so if they're training [AI on that]...*

[Unspecified] [44]

*I don't understand how it learns. If it learns wrong, who corrects it?*

[Participant 11] [37]

### The Impact of Physician-Patient Relationships

#### The Lack of Emotional Support and Human Interaction

While AI can provide more precise diagnoses and treatment recommendations on a technical level, patients emphasize that a physician’s emotional support and human interaction remain indispensable parts of the medical process. Especially when facing serious illnesses, patients require not only medical treatment but also emotional support from their physicians. AI cannot comprehend patients’ emotional fluctuations, anxieties, or unease. This lack of emotional resonance unsettles many patients. They believe that while AI can assist in treatment, it cannot replace the emotional care provided by health care professionals—a crucial element in the therapeutic process.

*People will still want human contact. Machines might be able to do a damned good job, but I think people still need that little human contact...*

[P_01] [28]

*Excessive digitalization reduces personal contact between people, and that, in turn, reduces communication and connection...*

[P_04] [27]

#### Challenges in Physician-Patient Communication

Patients widely agree that effective communication is fundamental to building trust between physicians and patients. Face-to-face interaction helps physicians better understand patients’ conditions while making patients feel respected and cared for. With AI integration, patients worry physicians may overrely on technology, reducing face-to-face interactions. They emphasize that while AI improves efficiency, it risks neglecting deep patient engagement, which is essential for building trust and understanding in the physician-patient relationship. Especially during complex disease treatments, patients hope physicians will listen more to their needs and feelings rather than solely relying on algorithmic decisions.

*It is crucial that he listen to me and that I listen to him. That’s really the most important thing...* [27]

*I wouldn't only rely on Cronko [AI]; I would use Cronko alongside a physician...*

[P_13] [27]

### Trust and Accountability

#### Responsibility for AI Decision-Making

Patients widely express concern about unclear accountability when AI errors occur. AI decisions often lack the experience and emotional judgment of human physicians, making it difficult for patients to determine responsibility in the event of medical incidents. While acknowledging AI’s potential to enhance efficiency and accuracy, patients indicate that the absence of a clear accountability mechanism fosters significant distrust toward AI. Particularly when AI systems malfunction, patients are uncertain whether responsibility lies with developers, hospitals, or health care providers, creating unease about adopting AI-driven medical technologies. Additionally, patients emphasize the right to question and correct AI judgments. They argue that when AI makes decisions based on flawed data or assumptions, patients should have avenues to raise objections and demand corrections, rather than passively accepting machine conclusions:

*I believe the doctor always has the responsibility to be checking for you, and you're his responsibility, you know? The AI is not responsible; that’s just a tool.*

[Unspecified] [44]

*So I'd rather know what they're observing, and if it’s [AI] wrong, I would [want to be able to] correct it rather than have them just collect data and make assumptions.*

[Unspecified] [44]

#### Acceptance Based on Professional Trust

Despite reservations about AI, patients accept it when recommended by their medical team due to trust in their physician or health care institution. Patients believe that the medical team’s professional endorsement and experience enhance trust in AI systems. Many patients indicated that they would be more receptive if the medical team confirmed that the AI technology is thoroughly validated and offers benefits. Patients view physician recommendations and oversight as the primary basis for trusting AI technology. The professional team’s opinion serves as a safety net for patients, particularly when emerging technologies remain unproven.

*I trust it pretty much because I did ask how long they [the clinical team] have been doing LVAD, and I was surprised to find out that this had been going on longer than I thought it was...* [29]

### Ethical Challenges of AI and Health Care Equity

#### Requirement of Ethical Standards

Patients believe that the application of AI in health care requires strict ethical standards, particularly when handling sensitive data. The absence of reasonable regulations may compromise patient rights. Many patients worry that AI research may prioritize speed over ethical standards, leading to data misuse or privacy violations. Additionally, patients emphasize the right to informed consent regarding AI interventions. Some patients believe health care institutions should disclose AI use before consultations, not during them, allowing time to process information and prepare mentally. Therefore, AI technology must operate under rigorous ethical review and legal frameworks to safeguard patients’ fundamental rights:

*I am afraid that [...] those who do research would like to reach a result...[but] the result should be so well grounded that you can actually vouch for it all the way.*

[M30] [30]

*I want to know about it [AI involvement] before I get to the doctor... I’d rather have the opportunity to think about it and review it beforehand.*

[Participant] [45]

#### Threats to Health Care Equity

AI’s impact on health care equity presents a complex duality. Some patients fear AI may exacerbate existing inequalities—low-income groups and the technologically disadvantaged may struggle to access AI-enabled health care equitably, thereby widening health disparities. However, evidence from marginalized communities reveals another possibility: minority patients view AI as a “safe space” enabling them to discuss sensitive issues without cultural shame, while multilingual capabilities compensate for dialect service gaps and bypass human “gatekeepers” perceived as biased.

*At that moment, I didn't want to speak to anyone... If I had this tool, I would use it first before speaking to anyone else.*

[P01, male, aged 28 y] [39]

*I feel that patients with fewer resources or access to technology will fall behind in terms of benefiting from AI in health care.*

[Unspecified] [26]

### The Irreplaceability of Human Care

Patients believe that while AI can provide technical support, it cannot replace the human care and ethical judgment physicians offer in treatment. AI cannot comprehend patients’ emotional needs or life contexts, factors crucial to treatment decisions. Patients emphasized that AI should only serve as an auxiliary tool, unable to substitute for physicians’ roles in complex medical decisions—particularly regarding emotional support and personalized treatment.

*Humans can express emotions, empathy, help, and give hope for a better tomorrow better than any machine.*

[P_04] [27]

### Future Outlook for AI Applications

#### AI Technology Diffusion

Patients believe that the advancement of AI in health care is inevitable. Despite concerns, they acknowledge AI’s potential to enhance efficiency and reduce errors, particularly in areas such as diagnostics, treatment planning, and patient management. As AI becomes widely adopted across global industries, patients recognize that it will become a vital component of future health care—although privacy and ethical issues arising from the technology require further regulation to ensure that patient data are protected and that AI systems are used responsibly in clinical settings.

*It’s becoming more and more the case that robots are controlling much of everyday life in various professions, and it’s happening everywhere.*

[M12] [30]

#### Potential for AI-Physician Collaboration

Patients hold a positive view of AI collaborating with physicians, believing it can alleviate physicians’ workload and improve efficiency, but it cannot replace the physician’s decision-making role. Patients hope AI serves as an auxiliary tool to assist physicians rather than dominate the treatment process. Especially in complex conditions, the physician’s clinical judgment and humanistic care remain crucial.

*I think the whole system makes sense, but it can’t function independently.*

[P_13] [27]

## Discussion

### Principal Findings

This study aimed to conduct an in-depth analysis of patients’ ethical concerns regarding AI medical applications, explore their impact on patient acceptance and trust, and use SET to reveal multilevel tension mechanisms. SET, which is based on Bronfenbrenner’s ecological systems theory [51], was later improved by researchers such as McLeroy and has become a well-known way to study public health and health behavior [52,53]. The theory’s core assumption posits that individual behavior does not exist in isolation but is embedded within a multilevel, interacting environmental system. The microlevel focuses on individual knowledge, attitudes, and psychological characteristics; the mesolevel involves interpersonal relationships and organizational environments; and the macrolevel encompasses sociocultural norms and policy systems [52,54]. These 3 levels form a complex ecosystem through dynamic bidirectional influences, where changes in any one level may propagate to others.

Through a meta-synthesis of 25 qualitative studies, we identified 6 core themes: privacy and data security, technological reliability, impacts on physician-patient relationships, trust and accountability, ethical challenges and health care equity, and future perspectives. Socioecological analysis reveals that these concerns create mutually reinforcing “ecological imbalances” across micro (technological cognitive biases and data control anxieties), meso (broken physician-patient trust and institutional accountability gaps), and macro (health inequities and lagging ethical standards) levels, thereby hindering patient acceptance and trust in AI technologies. Using this framework, the following sections will examine these findings in detail.

### Microlevel: The Vicious Cycle of Technological Cognitive Bias and Data Control Anxiety

At the individual level, patients’ concerns about AI technology primarily manifest as cognitive uncertainty about the technology and anxiety over data control. SET emphasizes the interaction between individual behavior and multilevel environments, where individuals’ cognitive and emotional attitudes are influenced not only by internal factors but also closely tied to their social and cultural contexts [55]. During the application of AI medical technologies, individuals’ cognitive biases about the technology are often accompanied by intense concerns about its transparency and controllability, which can lead to hesitance in adopting these technologies for their health care needs.

Research indicates that patients’ understanding of AI technology is closely linked to their acceptance of it [56]. However, the complexity of AI technology and the black box nature of algorithms make it difficult for patients to comprehend its decision-making processes. This uncertainty directly leads to patients questioning the technology’s accuracy [57]. For instance, patients worry that AI cannot account for their individualized health variations—such as differences in underlying conditions or lifestyle habits—potentially compromising the effectiveness of medical interventions [58]. Furthermore, patients’ doubts about AI accuracy have extended from the algorithmic level to the data level. Drawing from experiences reviewing their own electronic health records, they have identified numerous errors in the documentation. Consequently, they fear that even well-designed algorithms trained on such flawed data may struggle to produce reliable outputs. This technological cognitive bias does not exist in isolation; it is intertwined with individuals’ anxieties over data control. Patients’ concerns about data control cannot be reduced to instrumental worries about privacy breaches but should be understood as existential threats to their self-integrity and narrative sovereignty [59]. Patients’ anxiety over data control extends beyond privacy leaks to a profound fear of losing self-determination [60]. Medical data encapsulate patients’ life experiences, health statuses, and identity information. When processed by algorithms for unforeseen purposes, patients lose not only informational control but also dominion over their own health narratives [61-63].

SET posits that individual behavior is shaped by multiple interrelated factors [55]. At the microlevel, patients’ technological cognitive biases and data control anxieties do not exist in isolation but form a vicious cycle through the “cognition-anxiety” interaction [64]. Distrust of AI technology not only heightens patients’ concerns about data security but also deepens their skepticism regarding technological transparency and controllability [65]. This vicious cycle may lead to patient resistance toward AI technology, subsequently affecting their overall attitude toward health care services. Similar perspectives are reflected in other studies. Research indicates that uncertainty about information technology and privacy concerns often amplify individual resistance, thereby influencing their acceptance of technology [66].

### Mesolevel: Resonance Between Fractured Physician-Patient Trust and Institutional Accountability Deficits

At the mesolevel, patient concerns primarily manifest as fractures in physician-patient trust and inadequacies in health care organizational governance. Interpersonal relationships and organizational culture often constrain individual behavioral changes, according to SET [55]. Patients’ apprehensions toward AI medical technologies stem not only from individual perceptions of the technology but are also closely intertwined with the quality of physician-patient relationships and the accountability of health care organizations.

Extensive research indicates that emotional empathy and interpersonal interaction between physicians and patients are crucial for building trust [67-69]. However, the introduction of AI has undermined this foundation to some extent. Studies reveal that when physicians overrely on AI technology, patients experience reduced interaction time and emotional support from their physicians, leading to diminished trust [70]. The core of the physician-patient relationship lies in physicians viewing patients as whole individuals, not machines requiring repair [71]. This relationship is based on listening, understanding, and being there for each other. Physicians know how much their patients are hurting and help them by talking to them [72-74]. AI intervention threatens the quality of this interpersonal engagement. When physicians’ attention shifts to screens, when diagnoses rely on algorithmic outputs, and when communication is replaced by standardized processes, patients transform from “people receiving care” into “objects undergoing testing” [70,75].

Conversely, the absence of accountability mechanisms within health care organizations is another significant source of patient concern. Existing research indicates that many institutions lack clear AI liability frameworks, leaving patients unable to identify responsible parties when AI systems malfunction [76]. This lack of accountability not only makes patients less trusting of AI technology but also makes health care organizations look less credible [77]. SET emphasizes that deficiencies in internal organizational governance structures amplify individual-level concerns by eroding patients’ trust in the health care system [78]. The absence of accountability in AI decision-making within health care organizations leaves patients without effective avenues for redress when encountering medical issues, thereby creating a mutually reinforcing negative cycle of “accountability-trust” [79]. Similar “accountability vacuum” issues have been explored in other studies, with research indicating that patients’ trust in medical decisions is often severely compromised when health care organizations lack clear responsibility frameworks [80].

### Macrolevel: Intensifying Issues of Health Care Equity and Lagging Ethical Standards

At the macrolevel, patient concerns regarding AI technology primarily center on health care equity and the lag in ethical regulations. SET asserts that sociocultural inequities and the lack of ethical standards directly impact individual behavioral choices [81]. During the implementation of AI health care technologies, widespread adoption encounters obstacles arising from socioeconomic disparities. Low-income groups and patients with limited technological literacy often struggle to access AI medical services equitably. This gap exacerbates health care inequalities, fueling patient resistance toward AI technologies [82,83].

Not all socioeconomic strata equally benefit from the application of AI health care technologies, especially in regions with slower economic development or scarce resources [84]. High-end medical facilities and advanced technological resources are concentrated in major cities and economically developed regions, while low-income communities and remote areas still face significant gaps in AI health care adoption [85]. Furthermore, the high technical support and maintenance requirements of AI health care technologies make them unaffordable for many resource-constrained medical institutions, further exacerbating the unequal distribution of health care resources [86]. In this context, the “technological access barriers” experienced by patients are not merely technical difficulties but deep-seated social problems stemming from unequal socioeconomic structures and resource allocation [87].

Although the World Health Organization (WHO) has issued global AI ethics principles (WHO, 2021), implementation and regulatory rigor vary significantly across countries [88]. The lag in ethical standards manifests not only at the policy level but also creates gaps in practical implementation. For instance, some countries may lack cross-regional ethical collaboration mechanisms, leading certain health care institutions to prioritize AI services for economically advantaged groups over low-income populations due to cost considerations [89]. Such practices exacerbate health inequalities, deepening patients’ concerns that AI serves only select groups rather than benefiting the broader public [90].

### Interaction Mechanisms and Mutual Influence Across Levels

According to SET, concerns at the microlevel, mesolevel, and macrolevel do not exist in isolation but form mutually reinforcing chain reactions through “risk perception transmission” [55]. Research indicates that individual cognition and anxiety at the microlevel generate reverse effects at interpersonal and organizational levels, subsequently impacting broader social structures [91]. At the microlevel, individuals’ cognitive biases regarding AI technology and anxieties over data control rights are transmitted to the mesolevel through interactions between individuals and health care organizations, increasing communication pressures for these organizations when deploying AI technologies [92,93]. Patients’ concerns about data privacy and security influence individual behavioral decisions and prompt health care institutions to reevaluate the boundaries of AI applications, thereby driving societal-level attention to AI ethical norms [94].

The absence of accountability mechanisms for medical organizations at the mesolevel exacerbates microlevel cognitive biases and anxieties, leading to increased distrust among patients and further complicating the integration of AI technologies in health care [93,95]. Extensive research indicates that the breakdown of social support networks directly impacts individual health decisions and attitudes, with trust deficits further intensifying emotional distress and technological apprehension [96-98]. The breakdown of physician-patient trust not only reduces patient acceptance of AI technology but also leads patients to rely more on personal emotional judgments when facing medical decisions, overlooking the potential of AI technology [99].

Social equity issues at the macrolevel further permeate the microlevel, particularly the rejection of AI health care services by low-income groups. This affects their acceptance of AI technology and reinforces fears of technological displacement through sociocultural perceptions [17,100]. SET indicates that sociocultural beliefs not only shape patients’ perceptions of AI technology through individual behavior but also influence organizational behavior via mesolevel cultural diffusion, thereby amplifying implementation barriers in unequal societies [101,102].

### Policy Recommendations: Specific Pathways and Response Strategies

Reflecting on the findings of this study, patients’ concerns are far from unfounded. Across the 25 studies included in this review, patients from diverse clinical contexts and cultural backgrounds consistently expressed anxieties about data security, skepticism toward algorithmic opacity, and questions about accountability attribution. These concerns point to objective limitations of AI medical technologies at their current stage of development and institutional gaps that remain unaddressed. Recent studies have independently corroborated the reality of these issues from various perspectives: data bias and privacy risks in clinical AI systems have been extensively documented [103], the erosion of physician-patient trust caused by algorithmic inexplicability has attracted sustained attention [104], and accountability attribution in AI-assisted medical decision-making still lacks clear legal delineation [105]. The WHO, in its 2021 guidance, also identified transparency, accountability, and equity as core principles for the ethical governance of AI in health care [106]. In other words, the concerns articulated by patients based on their lived experiences correspond precisely with the risks identified through systematic analyses by the academic community and international organizations. This implies that the goal of policy intervention should not be to “correct patient misconceptions,” but rather to substantively address these well-founded and evidence-based concerns. The following recommendations are organized across the microlevel, mesolevel, and macrolevel while acknowledging the implementation challenges inherent in each pathway.

### Microlevel: Incremental Transparency and Patient Empowerment

Given that the field of Explainable Artificial Intelligence remains in its developmental stages, fully visualizing algorithmic decision-making logic is not realistic in the short term; an incremental transparency strategy should therefore be adopted [107]. Specifically, drawing on the access rights for data subjects under the European Union’s General Data Protection Regulation [108], health care institutions should be required to establish data access logging systems that enable patients to query the access records and intended purposes of their health data [109]. To address patients’ concerns regarding the quality of training data, accessible mechanisms for medical record review and correction should be established, safeguarding patients’ rights to audit and amend their own medical records [110]. Furthermore, informed consent regarding AI involvement should be front-loaded—patients should be notified before their clinical encounter—and channels for questioning AI judgments and requesting human review should be established [111].

### Mesolevel: Rebuilding Trust and Clarifying Accountability

The principle of “human oversight, AI assistance” should be institutionally safeguarded. Rather than imposing rigid communication time benchmarks, mandatory “physician confirmation checkpoints” should be embedded within AI-assisted diagnostic and treatment workflows, ensuring that critical decisions are subject to physician review and explained to patients before implementation [112]. Concurrently, performance evaluation systems should be adjusted to prevent efficiency-driven metrics from encroaching upon the space for physician-patient communication [113]. Regarding accountability attribution, a tiered liability framework should be constructed that differentiates the responsible parties for data errors, algorithmic defects, and clinical misjudgments [114], while acknowledging that existing legal frameworks contain gaps in the attribution of responsibility for AI-driven decisions, necessitating legislative follow-up [115]. Additionally, clinical scenarios in which AI cannot substitute for human practitioners should be explicitly delineated, particularly those highly dependent on emotional support and clinical judgment, such as end-of-life care, mental health treatment, and complex decision-making situations that require empathy and nuanced understanding of patient needs [116].

### Macrolevel: Advancing Equitable Access and Ethical Governance

The WHO has set out global ethical standards for AI in health care, which can be used to help create an international ethical framework. National governments should build upon this foundation to develop regulatory frameworks aligned with their respective health care systems and cultural traditions, rather than pursuing an unrealistic goal of globally uniform standards. At the level of resource allocation, investment should be increased for low-income populations and remote areas, and age-friendly and low-cost AI health care tools should be developed to narrow the gap in technological accessibility [117]. However, it must be acknowledged that the barriers facing these regions extend beyond equipment scarcity to include insufficient technical maintenance capacity and digital health literacy, necessitating complementary capacity building and educational support [118]. Furthermore, equity assessments should be incorporated into the market approval review of AI health care products, requiring developers to submit impact assessment reports for diverse population groups [119].

Finally, it must be acknowledged that structural tensions exist in the advancement of AI in health care: technology developers’ pursuit of algorithmic efficiency and commercial returns may conflict with patients’ safety needs; health care institutions under cost pressures may find it difficult to reconcile these with adequate physician-patient communication. Confronting rather than evading these tensions is a prerequisite for formulating pragmatic policies [120].

### Research Limitations

This study has the following limitations. First, the 25 included studies were primarily conducted in developed regions, such as North America, Europe, and Australia, with insufficient representation of patients from developing countries. This limits the generalizability of findings to resource-constrained settings. Second, the included studies covered a wide range of AI applications—including imaging diagnostics, clinical decision support, and virtual health assistants—where patient concerns may vary across different AI types. The thematic synthesis in this study may obscure such context-specific differences. Third, as a secondary analysis, the quality of the meta-synthesis depends on the reporting depth of the original studies. Some studies provided insufficient contextual information for patient quotes, hindering more nuanced interpretation. Finally, AI medical technology is undergoing rapid iteration. The literature included in this study reflects patient perceptions within a specific time window. As technological transparency and regulatory frameworks evolve, patient attitudes may change, potentially leading to increased trust in AI medical technology and greater acceptance of its use in health care. Future research should continuously track this dynamic process.

### Conclusions

This research, through a systematic synthesis of 25 qualitative studies, identified 6 primary patient concerns regarding AI health care applications: privacy and data security, technological reliability, the impact on physician-patient relationships, trust and accountability, ethics and fairness, and ambivalent attitudes toward future developments. Unlike previous reviews focusing on the general public, this study centers on patients as core stakeholders. It pioneers the application of SET to this field, revealing a “disrupted ecological equilibrium” mechanism that propagates across microlevel, mesolevel, and macrolevel. This provides an explanatory framework—transcending descriptive induction—for understanding the deep-seated reasons behind patient resistance to AI medical technologies. The findings offer direct implications for practice: clinical institutions should establish a “human-led, AI-assisted” diagnostic model; policymakers should accelerate liability legislation and prioritize equitable technology access; and developers should adopt incremental transparency strategies while providing patients with avenues for questioning and review. Future research may explore the following directions: incorporating perspectives from patients in more developing countries and resource-constrained regions to test cross-cultural applicability; conducting comparative studies across different AI application scenarios to explore the context-specific nature of concerns; and using longitudinal designs to track the dynamic evolution of patient attitudes as technology advances and regulatory frameworks mature.

## Supplementary material

10.2196/85663 Multimedia Appendix 1 Search stage.

10.2196/85663 Checklist 1 PRISMA 2020 checklist.

10.2196/85663 Checklist 2 ENTREQ checklist.

10.2196/85663 Checklist 3 PRISMA-S checklist.

## Abbreviations

AI: artificial intelligence
ENTREQ: Enhancing Transparency in Reporting the Synthesis of Qualitative Research
GRADE-CERQual: Confidence in the Evidence from Reviews of Qualitative Research
JBI-QARI: Joanna Briggs Institute Qualitative Assessment and Review Instrument
PRISMA: Preferred Reporting Items for Systematic Reviews and Meta-Analyses
PRISMA-S: Preferred Reporting Items for Systematic Reviews and Meta-Analyses literature search extension
SET: social ecological theory
WHO: World Health Organization
