# Supplementary material for: Patient Concerns Regarding Artificial Intelligence Applications in Health Care: Systematic Review and Meta-Synthesis Based on Social Ecological Theory
Source: J Med Internet Res. 2026 Apr 28;28:e85663. doi: 10.2196/85663 (PMC13124089; doi:10.2196/85663)
Supplement: Multimedia Appendix 1 [file jmir-v28-e85663-s001.pdf]

Pubmed

#1 ("Artificial Intelligence"[MeSH]) OR ("Machine Learning"[MeSH]) OR ("Decision Support Systems, Clinical"[MeSH]) OR ("artificial intelligence"[tiab]) OR ("machine learning"[tiab]) OR ("deep learning"[tiab]) OR ("AI-based"[tiab]) OR ("AI-assisted"[tiab]) OR ("AI-driven"[tiab]) OR ("ChatGPT"[tiab]) OR ("large language model"[tiab]) OR ("clinical decision support"[tiab]) OR ("CDSS"[tiab]) OR ("generative AI"[tiab]) OR ("algorithm*"[tiab])

#2 ("Patients"[MeSH]) OR (patient*[tiab]) OR (stakeholder*[tiab])

#3 (healthcare[tiab]) OR ("health care"[tiab]) OR (medical[tiab]) OR (clinical[tiab])

#4 ("Attitude to Health"[MeSH]) OR (concern[tiab]) OR (perception[tiab]) OR (perspective[tiab]) OR (attitude[tiab]) OR (trust[tiab]) OR (acceptance[tiab]) OR (barrier[tiab]) OR (ethical[tiab]) OR (privacy[tiab]) OR (view[tiab]) OR (opinion[tiab]) OR (experience[tiab]) OR (feeling[tiab]) OR (worry[tiab])

#5 ("Qualitative Research"[MeSH]) OR (qualitative[tiab]) OR ("focus group"[tiab]) OR (interview[tiab]) OR ("thematic analysis"[tiab]) OR ("content analysis"[tiab]) OR ("grounded theory"[tiab]) OR (phenomenolog*[tiab]) OR (ethnograph*[tiab]) OR ("lived experience"[tiab]) OR ("narrative analysis"[tiab]) OR ("in-depth interview"[tiab]) OR ("qualitative study"[tiab]) OR ("qualitative research"[tiab])

#6 #1 AND #2 AND #3 AND #4 AND #5

Search	Actions	Details	Query	Results	Time
#6	•••	>	Search: **((((("Artificial Intelligence"[MeSH]) OR ("Machine Learning"[MeSH]) OR ("Decision Support Systems, Clinical"[MeSH]) OR ("artificial intelligence"[tiab]) OR ("machine learning"[tiab]) OR ("deep learning"[tiab]) OR ("AI-based"[tiab]) OR ("AI-assisted"[tiab]) OR ("AI-driven"[tiab]) OR ("ChatGPT"[tiab]) OR ("large language model"[tiab]) OR ("clinical decision support"[tiab]) OR ("CDSS"[tiab]) OR ("generative AI"[tiab]) OR ("algorithm*"[tiab])) AND (("Patients"[MeSH]) OR (patient*[tiab]) OR (stakeholder*[tiab]))) AND ((healthcare[tiab]) OR ("health care"[tiab]) OR (medical[tiab]) OR (clinical[tiab]))) AND (("Attitude to Health"[MeSH]) OR (concern[tiab]) OR (perception[tiab]) OR (perspective[tiab]) OR (attitude[tiab]) OR (trust[tiab]) OR (acceptance[tiab]) OR (barrier[tiab]) OR (ethical[tiab]) OR (privacy[tiab]) OR (view[tiab]) OR (opinion[tiab]) OR (experience[tiab]) OR (feeling[tiab]) OR (worry[tiab]))) AND (("Qualitative Research"[MeSH]) OR (qualitative[tiab]) OR ("focus group"[tiab]) OR (interview[tiab]) OR ("thematic analysis"[tiab]) OR ("content analysis"[tiab]) OR ("grounded theory"[tiab]) OR (phenomenolog*[tiab]) OR (ethnograph*[tiab]) OR ("lived experience"[tiab]) OR ("narrative analysis"[tiab]) OR ("in-depth interview"[tiab]) OR ("qualitative study"[tiab]) OR ("qualitative research"[tiab]))**	2,042	12:50:49
#5	•••	>	Search: **("Qualitative Research"[MeSH]) OR (qualitative[tiab]) OR ("focus group"[tiab]) OR (interview[tiab]) OR ("thematic analysis"[tiab]) OR ("content analysis"[tiab]) OR ("grounded theory"[tiab]) OR (phenomenolog*[tiab]) OR (ethnograph*[tiab]) OR ("lived experience"[tiab]) OR ("narrative analysis"[tiab]) OR ("in-depth interview"[tiab]) OR ("qualitative study"[tiab]) OR ("qualitative research"[tiab])**	697,188	12:50:31
#4	•••	>	Search: **("Attitude to Health"[MeSH]) OR (concern[tiab]) OR (perception[tiab]) OR (perspective[tiab]) OR (attitude[tiab]) OR** [illegible]	3,248,892	10:29:12

Embase

#1 ('artificial intelligence'/exp) OR ('clinical decision support system'/exp) OR ('artificial intelligence':ti,ab) OR ('machine learning':ti,ab) OR ('deep learning':ti,ab) OR ('clinical decision support':ti,ab) OR ('chatgpt':ti,ab) OR ('large language model':ti,ab) OR ('ai-based':ti,ab) OR ('ai-assisted':ti,ab) OR ('ai-driven':ti,ab) OR ('cdss':ti,ab) OR ('generative ai':ti,ab) OR ('algorithm*':ti,ab)

#2 ('patient'/exp) OR (patient*:ti,ab) OR (stakeholder*:ti,ab)

#3 (healthcare:ti,ab) OR ('health care':ti,ab) OR (medical:ti,ab) OR (clinical:ti,ab)

#4 ('attitude'/exp) OR (concern:ti,ab) OR (perception:ti,ab) OR (perspective:ti,ab) OR

(attitude:ti,ab) OR (trust:ti,ab) OR (acceptance:ti,ab) OR (barrier:ti,ab) OR (ethical:ti,ab) OR (privacy:ti,ab) OR (view:ti,ab) OR (opinion:ti,ab) OR (experience:ti,ab) OR (feeling:ti,ab) OR (worry:ti,ab)

#5 ('qualitative research'/exp) OR (qualitative:ti,ab) OR ('focus group':ti,ab) OR (interview:ti,ab) OR ('thematic analysis':ti,ab) OR ('content analysis':ti,ab) OR ('grounded theory':ti,ab) OR (phenomenolog*:ti,ab) OR (ethnograph*:ti,ab) OR ('lived experience':ti,ab) OR ('narrative analysis':ti,ab) OR ('in-depth interview':ti,ab) OR ('qualitative study':ti,ab) OR ('qualitative research':ti,ab)

#6 #1 AND #2 AND #3 AND #4 AND #5

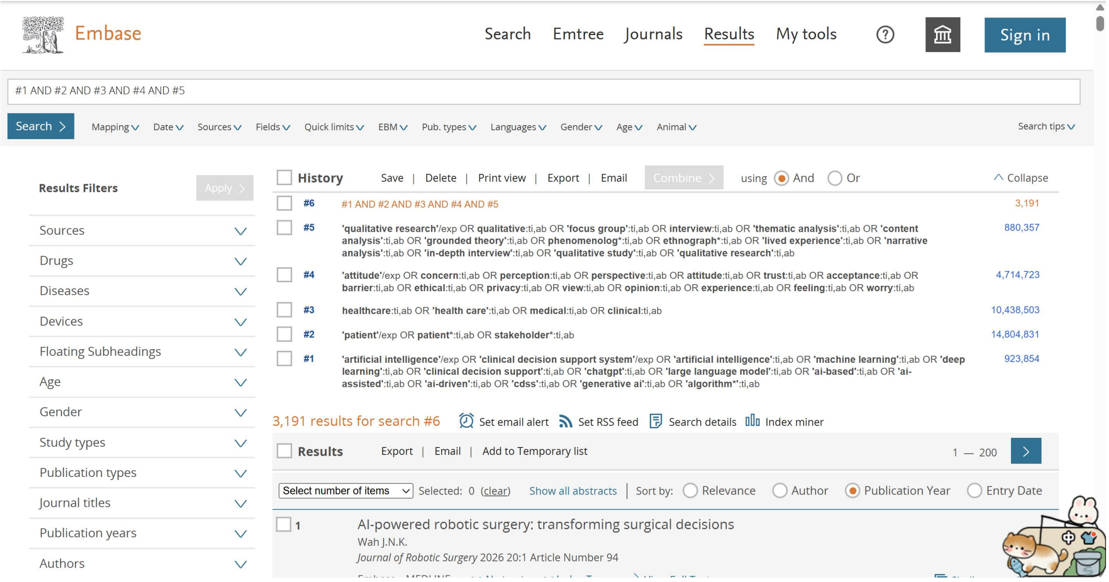

Web of Science

TS=("artificial intelligence" OR "machine learning" OR "deep learning" OR "AI-based" OR "AI-assisted" OR "AI-driven" OR "ChatGPT" OR "large language model" OR "clinical decision support" OR "CDSS" OR "generative AI" OR "algorithm*")

AND TS=(patient* OR stakeholder*)

AND TS=(healthcare OR "health care" OR medical OR clinical)

AND TS=(concern OR perception OR perspective OR attitude OR trust OR acceptance OR barrier OR ethical OR privacy OR view OR opinion OR experience OR feeling OR worry)

AND TS=(qualitative OR "focus group" OR interview OR "thematic analysis" OR "content analysis" OR "grounded theory" OR phenomenolog* OR ethnograph* OR "lived experience" OR "narrative analysis" OR "in-depth interview" OR "qualitative study" OR "qualitative research")

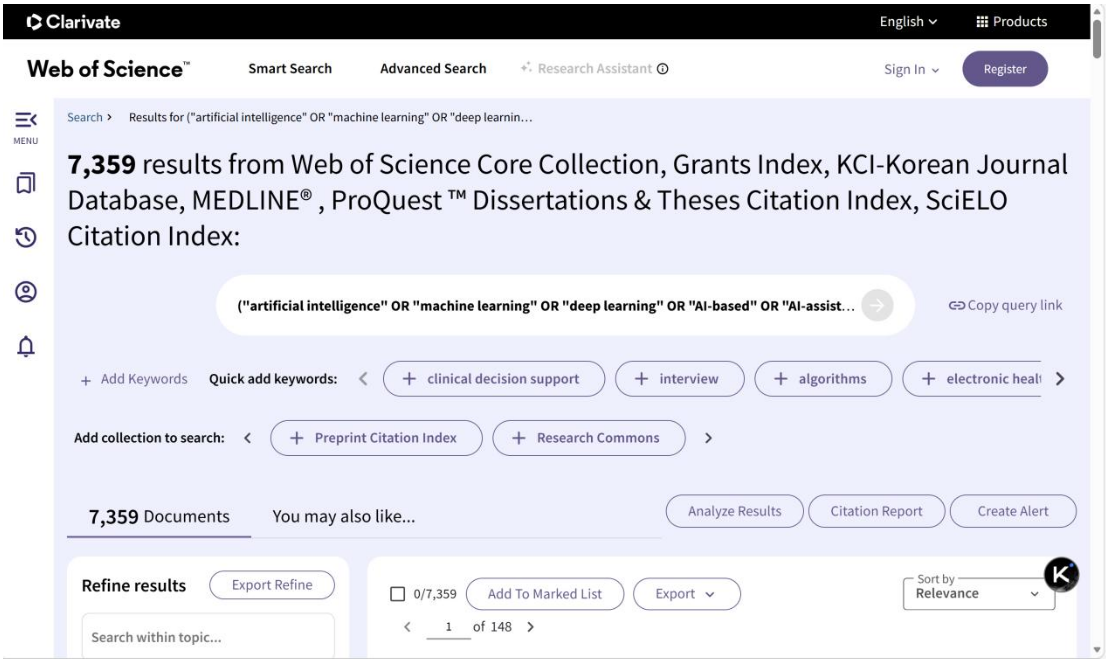

CINHAL Ultimate

("artificial intelligence" OR "machine learning" OR "deep learning" OR "AI-based" OR "AI-assisted" OR "AI-driven" OR "ChatGPT" OR "large language model" OR "clinical decision support" OR "CDSS" OR "generative AI" OR "algorithm*")
AND (patient* OR stakeholder*)
AND (healthcare OR "health care" OR medical OR clinical)
AND (concern OR perception OR perspective OR attitude OR trust OR acceptance OR barrier OR ethical OR privacy OR view OR opinion OR experience OR feeling OR worry)
AND (qualitative OR "focus group" OR interview OR "thematic analysis" OR "content analysis" OR "grounded theory" OR phenomenolog* OR ethnograph* OR "lived experience" OR "narrative analysis" OR "in-depth interview" OR "qualitative study" OR "qualitative research")

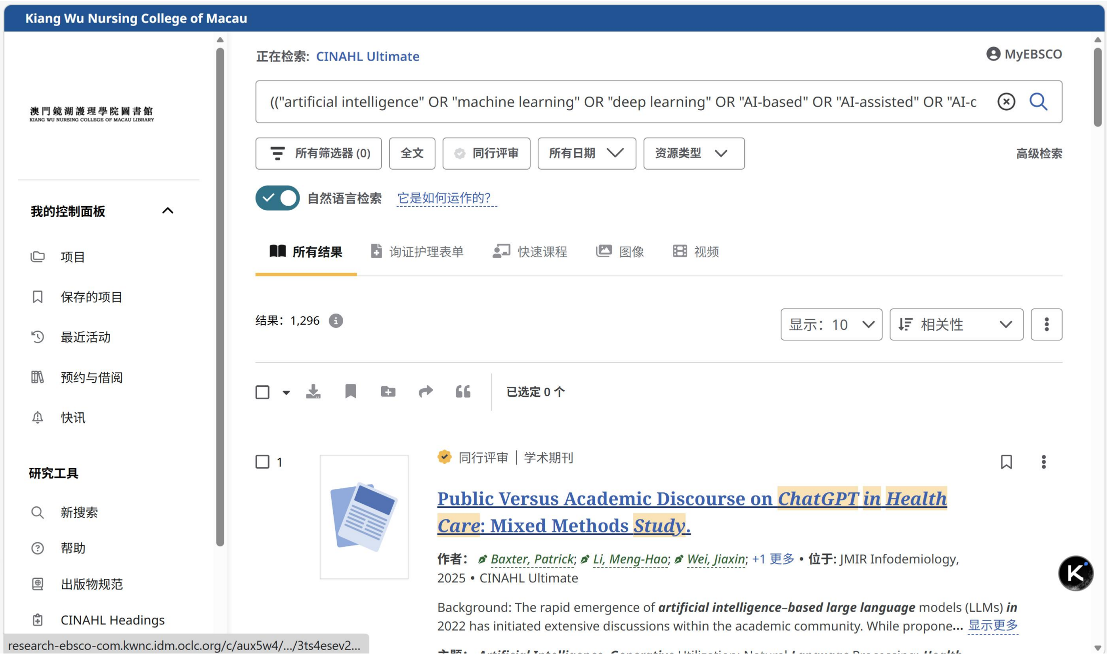

Scopus

TITLE-ABS-KEY("artificial intelligence" OR "machine learning" OR "deep learning" OR "AI-based" OR "AI-assisted" OR "AI-driven" OR "ChatGPT" OR "large language model" OR "clinical decision support" OR "CDSS" OR "generative AI" OR "algorithm*")

AND TITLE-ABS-KEY(patient* OR stakeholder*)

AND TITLE-ABS-KEY(healthcare OR "health care" OR medical OR clinical)

AND TITLE-ABS-KEY(concern OR perception OR perspective OR attitude OR trust OR acceptance OR barrier OR ethical OR privacy OR view OR opinion OR experience OR feeling OR worry)

AND TITLE-ABS-KEY(qualitative OR "focus group" OR interview OR "thematic analysis" OR "content analysis" OR "grounded theory" OR phenomenolog* OR ethnograph* OR "lived experience" OR "narrative analysis" OR "in-depth interview" OR "qualitative study" OR "qualitative research")

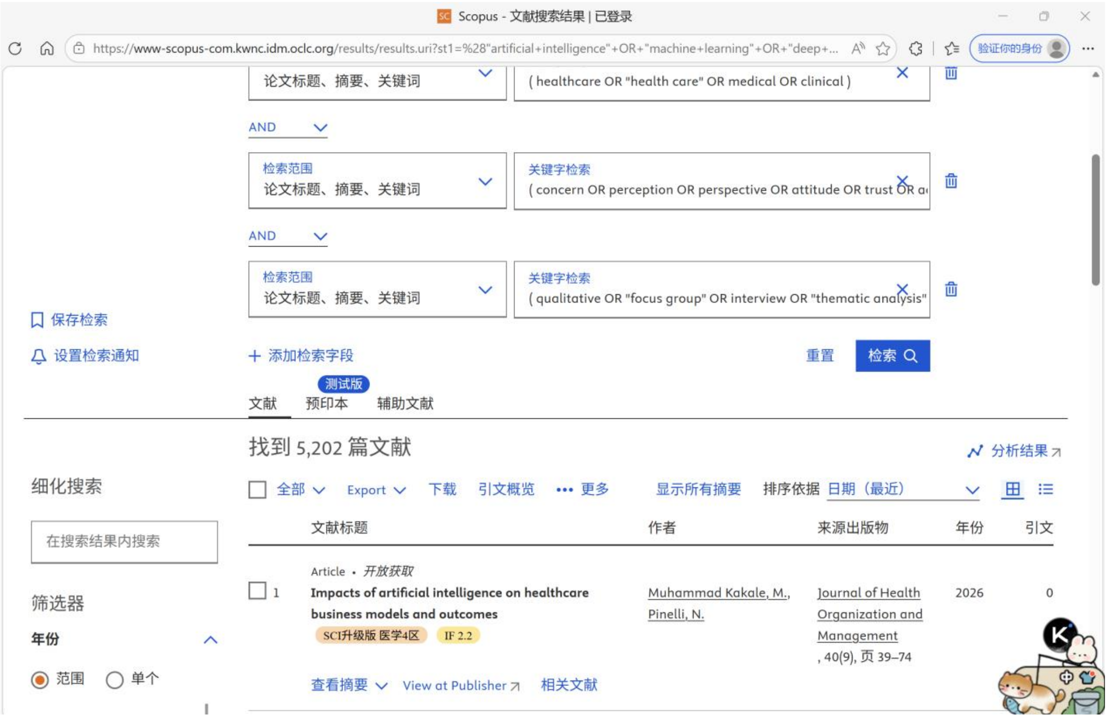
Scopus - 文献搜索结果 | 已登录
https://www-scopus-com.kwnc.idm.oclc.org/results/results.uri?st1=%28"artificial+intelligence"+OR+"machine+learning"+OR+"deep+...
验证你的身份
论文标题、摘要、关键词
(healthcare OR "health care" OR medical OR clinical)
AND
检索范围
论文标题、摘要、关键词
关键字检索
(concern OR perception OR perspective OR attitude OR trust OR a
AND
检索范围
论文标题、摘要、关键词
关键字检索
(qualitative OR "focus group" OR interview OR "thematic analysis"
保存检索
设置检索通知
添加检索字段
重置
检索
测试版
文献
预印本
辅助文献
找到 5,202 篇文献
分析结果
细化搜索
在搜索结果内搜索
全部
Export
下载
引文概览
更多
显示所有摘要
排序依据
日期（最近）
文献标题
作者
来源出版物
年份
引文
Article • 开放获取
1
Impacts of artificial intelligence on healthcare business models and outcomes
SCI升级版 医学4区
IF 2.2
Muhammad Kakale, M., Pinelli, N.
Journal of Health Organization and Management
, 40(9), 页 39–74
2026
0
筛选器
年份
范围
单个
查看摘要
View at Publisher
相关文献
