# Supplementary material for: Patient Concerns Regarding Artificial Intelligence Applications in Health Care: Systematic Review and Meta-Synthesis Based on Social Ecological Theory
Source: J Med Internet Res. 2026 Apr 28;28:e85663. doi: 10.2196/85663 (PMC13124089; doi:10.2196/85663)
Supplement: Checklist 3 [file jmir-v28-e85663-s004.pdf]

PRISMA-S Checklist

Section/topic	#	Checklist item	Location(s) Reported
**INFORMATION SOURCES AND METHODS**			
Database name	1	Name each individual database searched, stating the platform for each.	PubMed (via NLM), Embase (via Embase.com), Web of Science (via Clarivate), CINAHL (via EBSCOhost), and Scopus (via Scopus.com) (Methods, Search Strategy).
Multi-database searching	2	If databases were searched simultaneously on a single platform, state the name of the platform, listing all of the databases searched.	Web of Science (via Clarivate) simultaneously searched multiple databases including Core Collection, KCI-Korean Journal Database, MEDLINE, ProQuest Dissertations & Theses Citation Index, SciELO Citation Index, and Grants Index. All other databases were searched individually (Methods, Search Strategy).
Study registries	3	List any study registries searched.	Study registries were not searched, as this review focused on published qualitative studies rather than ongoing or unpublished trials (Methods, Search Strategy).
Online resources and browsing	4	Describe any online or print source purposefully searched or browsed (e.g., tables of contents, print conference proceedings, web sites), and how this was done.	No online browsing, conference proceedings, or print sources were purposefully searched beyond the databases and reference lists described (Methods, Search Strategy).
Citation searching	5	Indicate whether cited references or citing references were examined, and describe any methods used for locating cited/citing references (e.g., browsing reference lists, using a citation index, setting up email alerts for references citing	Reference lists of included studies were manually searched to identify additional relevant studies (Methods, Search

		included studies).	Strategy).
Contacts	6	Indicate whether additional studies or data were sought by contacting authors, experts, manufacturers, or others.	No additional studies or data were sought by contacting authors or experts (Methods, Search Strategy).
Other methods	7	Describe any additional information sources or search methods used.	No additional search methods were used beyond systematic database searching and reference list checking (Methods, Search Strategy).
**SEARCH STRATEGIES**			
Full search strategies	8	Include the search strategies for each database and information source, copied and pasted exactly as run.	Methods, Search Strategy; Supplementary Material
Limits and restriction s	9	Specify that no limits were used, or describe any limits or restrictions applied to a search (e.g., date or time period, language, study design) and provide justification for their use.	No date or study design limits were applied. Language was restricted to English and Chinese. No other restrictions were used (Methods, Search Strategy).
Search filters	10	Indicate whether published search filters were used (as originally designed or modified), and if so, cite the filter(s) used.	No published search filters were used (Methods, Search Strategy).
Prior work	11	Indicate when search strategies from other literature reviews were adapted or reused for a substantive part or all of the search, citing the previous review(s).	The search strategy was developed de novo for this review and was not adapted from a prior review (Methods, Search Strategy).
Updates	12	Report the methods used to update the search(es) (e.g., rerunning searches, email alerts).	The search strategy was iteratively refined and all database searches were rerun from inception on January 4, 2026 and March 1, 2026 (Methods, Search Strategy).
Dates of searches	13	For each search strategy, provide the date when the last search occurred.	The initial search was conducted on September 28, 2025. Searches were rerun on January 4, 2026 and March 1, 2026 (Methods, Search Strategy).
**PEER REVIEW**			
Peer	14	Describe any search peer review process.	Formal peer review of the search

review			strategy using a validated instrument was not conducted. The strategy was collaboratively developed by the research team, iteratively refined, and piloted against known relevant studies to confirm adequate retrieval (Methods, Search Strategy).
**MANAGING RECORDS**			
Total Records	15	Document the total number of records identified from each database and other information sources.	A total of 19,090 records were identified across five databases. After deduplication, 11,958 records remained for title and abstract screening (Methods, Inclusion and Exclusion Criteria; Figure 1).
Deduplicat ion	16	Describe the processes and any software used to deduplicate records from multiple database searches and other information sources.	Duplicate records were identified and removed using EndNote 21, supplemented by manual checking (Methods, Inclusion and Exclusion Criteria).

PRISMA-S: An Extension to the PRISMA Statement for Reporting Literature Searches in Systematic Reviews
Rethlefsen ML, Kirtley S, Waffenschmidt S, Ayala AP, Moher D, Page MJ, Koffel JB, PRISMA-S Group.
Last updated
February 27,
2020.
